# Calcium Oxalate Nephrolithiasis and Gut Microbiota: Not just a Gut-Kidney Axis. A Nutritional Perspective

**DOI:** 10.3390/nu12020548

**Published:** 2020-02-20

**Authors:** Andrea Ticinesi, Antonio Nouvenne, Giulia Chiussi, Giampiero Castaldo, Angela Guerra, Tiziana Meschi

**Affiliations:** 1 Geriatric-Rehabilitation Department, Azienda Ospedaliero-Universitaria di Parma, Via Antonio Gramsci 14, 43126 Parma, Italy; anouvenne@ao.pr.it (A.N.); gchiussi@ao.pr.it (G.C.); castaldog@ao.pr.it (G.C.); angela.guerra@unipr.it (A.G.); tiziana.meschi@unipr.it (T.M.); 2 Microbiome Research Hub, University of Parma, Parco Area delle Scienze 11/A, 43124 Parma, Italy; 3 Department of Medicine and Surgery, University of Parma, Via Antonio Gramsci 14, 43126 Parma, Italy

**Keywords:** urolithiasis, diet, oxalate, renal calculi, microbiome, *Oxalobacter*

## Abstract

Recent studies have shown that patients with kidney stone disease, and particularly calcium oxalate nephrolithiasis, exhibit dysbiosis in their fecal and urinary microbiota compared with controls. The alterations of microbiota go far beyond the simple presence and representation of *Oxalobacter formigenes*, a well-known symbiont exhibiting a marked capacity of degrading dietary oxalate and stimulating oxalate secretion by the gut mucosa. Thus, alterations of the intestinal microbiota may be involved in the pathophysiology of calcium kidney stones. However, the role of nutrition in this gut-kidney axis is still unknown, even if nutritional imbalances, such as poor hydration, high salt, and animal protein intake and reduced fruit and vegetable intake, are well-known risk factors for kidney stones. In this narrative review, we provide an overview of the gut-kidney axis in nephrolithiasis from a nutritional perspective, summarizing the evidence supporting the role of nutrition in the modulation of microbiota composition, and their relevance for the modulation of lithogenic risk.

## 1. Introduction

The gastrointestinal system plays a pivotal role in the pathophysiology of idiopathic calcium oxalate nephrolithiasis, the most common form of kidney stone disease [1,2,3]. Gut mucosa absorption consistently influences both calcium and oxalate metabolism and represents a fundamental driver of hypercalciuria and hyperoxaluria, the two most important pro-lithogenic urinary metabolic abnormalities found in calcium oxalate stone formers [4,5,6]. The concepts of “absorptive hypercalciuria” and “enteric hyperoxaluria” imply the presence of a cross-talk between the gut and the kidney contributing to the pathophysiology of calcium oxalate stones [4,5,6]. 

The role of gut microbial communities, i.e., the microbiota, in these mechanisms remained uncertain until a few years ago [7]. The research was, in fact, mainly focused on only one component of the human gut microbiota, *Oxalobacter formigenes* [7]. The oxalate-degrading capacity of this Gram-negative anaerobic bacterium led to the assumption that calcium oxalate nephrolithiasis was associated with intestinal depletion of *Oxalobacter* [8]. Conversely, probiotic intervention with *Oxalobacter* or other species engineered with oxalate-degrading functionalities was believed to reduce the lithogenic risk [9,10]. Unfortunately, both observational and intervention studies gave conflicting results, leaving great uncertainty on the role of the microbiota in lithogenesis [7]. 

In the last decade, advanced omics techniques have allowed deep sequencing and functional characterization of gut microbial communities at an unprecedented level [11]. Thus, the early physiological concept of the gut-kidney axis in nephrolithiasis has been brushed up in light of the so-called microbiota revolution [7]. Recent studies have shown that calcium kidney stone formers have a different fecal microbiota composition than stone-free individuals, supporting the hypothesis that the microbiota is a major player in the pathophysiology of nephrolithiasis [7,12,13]. 

These studies have shed light on the gut-kidney axis in nephrolithiasis, but in most cases, failed to provide integration with clinical aspects of nephrolithiasis, and particularly nutrition. Nutritional imbalances, such as poor hydration, high salt, and animal protein and low calcium, fruit and vegetable (FAV) intake, are considered the main risk factors for calcium oxalate kidney stone disease [14,15]. Conversely, water therapy, adequate consumption of dairy products, FAVs, and low-salt low-animal protein diets are considered the pillars of non-pharmacological prevention of nephrolithiasis [16,17]. It is still uncertain how these well-established clinical concepts can be integrated into the novel microbiome-centered acquisitions on the gut-kidney axis, despite the fact that dietary habits are well-known determinants of gut microbiota composition. 

The aim of this narrative review is thus to summarize the current knowledge on the relationship between gut microbiota and calcium oxalate kidney stone disease from a nutritional perspective. 

## 2. Gut Microbiota and Calcium Oxalate Stone Disease: An Overview

### 2.1. Before the Microbiota Revolution: Focus on Oxalobacter 

*Oxalobacter formigenes* was isolated for the first time in 1980 from the rumen of some mammals and metabolically characterized as having a strong oxalate-degrading capacity [18]. It remains the most efficient oxalate-degrading biological system known to date, thanks to the expression of two enzymes, oxalyl-CoA decarboxylase, and formyl-CoA transferase, that allow the production of the soluble compound formate and CO_2_, with the release of energy that is used by the bacterium for cellular activities [19,20]. In the following years, *Oxalobacter* was isolated from the intestine of several mammals, including humans, and cultured on oxalate-rich mediums [21]. An inverse relationship between *Oxalobacter* presence in the intestinal lumen and oxalate absorption was also demonstrated in guinea pigs [22]. 

However, the possible role of *Oxalobacter* in human kidney stone disease was not further investigated until the late 1990s, when a polymerase chain reaction (PCR)-based method of *Oxalobacter* identification and quantification was developed [23]. *Oxalobacter* was detected in 30–70% of stool samples of humans, and its presence was significantly associated with high dietary oxalate intake and with reduced fractional absorption of oxalate [24]. The clinical significance of *Oxalobacter* in modulating lithogenic risk was, therefore, investigated. *Oxalobacter* may, in fact, protect against calcium nephrolithiasis through two distinct mechanisms: oxalate degradation in the gut lumen with reduction of mucosal absorption and promotion of endogenous oxalate secretion by the gut mucosa [25]. 

Observational studies conducted with cultural and PCR-based methodology showed that *Oxalobacter* colonization in fecal samples was significantly lower in stone formers, or patients with high lithogenic risk, than stone-free controls (Table 1) [26,27,28,29,30]. In idiopathic stone formers, a significant correlation between the status of *Oxalobacter* colonization and 24-h urinary oxalate excretion was detected in one study [30], but not in another [29]. Such a correlation was instead found in subjects at high risk of nephrolithiasis due to cystic fibrosis [26] or inflammatory bowel disease [28], but not in the morbidly obese [31]. The relationship between colonization status and oxaluria may depend on dietary oxalate intake, becoming more evident in experimental conditions under controlled dietary regimens [32].

Recent population-based studies combining the traditional species-specific microbiological techniques with metagenomics have highlighted that *Oxalobacter* is stably present in the fecal microbiome of only 31% of healthy young people living in the US [33]. This prevalence is much lower than that detected in tribal populations from Venezuela and Tanzania, supporting a possible role of diet and lifestyle in establishing *Oxalobacter* colonization [34]. In a large group of samples from the American Gut Project, the main factors associated with *Oxalobacter* colonization in gut microbiota were ethnicity, country of residence, older age, level of education, recent exposure to antibiotics, body weight, alcohol, and FAV intake [35]. A healthy lifestyle and nutrition may thus favorably influence gut microbiota composition towards stable colonization by *Oxalobacter*. 

Since the late 1990s, several intervention studies have investigated whether the administration of *Oxalobacter* or other probiotic blends engineered with oxalate-degrading functionalities could result in the reduction of lithogenic risk (Table 2) [36,37,38,39,40,41,42,43]. All these studies were conducted on small samples (the largest one having enrolled only 42 participants) and were highly heterogeneous for the type and duration of the intervention and for the clinical characteristics of participants, ranging from healthy volunteers to children with severe forms of primary hyperoxaluria (Table 2). The results were conflicting overall, with some studies reporting significant reductions in urinary oxalate excretion after probiotic treatment [36,37,38,40,41,42], and others showing no changes from baseline [39,43] (Table 2). The clinical significance of detected reductions in urinary oxalate excretion was also uncertain, since oxalate excretion is a surrogate outcome of stone recurrence, and only one of many elements concurring to the definition of lithogenic risk.

### 2.2. Beyond the Microbiota Revolution: Oxalobacter as Part of a Network

To date, the fecal microbiota composition of calcium stone formers has been investigated with next-generation sequencing techniques in seven different studies [44,45,46,47,48,49,50], summarized in Table 3. Two studies also investigated urinary microbiota composition comparing it with the microbiota of stones [50,51]. 

All these studies support the concept that gut microbiota dysbiosis, i.e., reduction of overall biodiversity with alteration of physiologic composition, is present in stone formers [44,45,46,47,48,49,50]. In recurrent stone formers with hyperoxaluria, Suryavanshi and colleagues found an increased representation of pathobionts and species with oxalate-degrading capacity, including *Oxalobacter*, compared with the controls [44]. Several of these taxa co-occurred in bacterial networks identifying different microbiota compositions between stone formers and controls. Most notably, *Prevotella, Dialister,* and *Faecalibacterium* were depleted in stone formers, while *Bacteroides* were overrepresented [44]. 

The concept of gut microbiota dysbiosis associated with nephrolithiasis was later confirmed by two small-sized studies conducted on heterogeneous groups of stone formers [45,46] and by a larger case-control study conducted on recurrent idiopathic calcium oxalate stone formers [47]. In that study, the differences of gut microbiota composition between stone formers and controls were independent of body composition, diet, hydration, urinary factors of lithogenic risk, and bowel movements, and included reduced representation of some key taxa for the maintenance of eubiosis, such as the short-chain fatty acid (SCFA) producer *Faecalibacterium prausnitzii* [47]. Moreover, the oxalate degrading capacity of the microbiota, inferred by shotgun metagenomics sequencing, was higher in controls, with the average abundance of several bacterial taxa that were inversely correlated with urinary oxalate excretion [47], a finding that was also coherent with the Suryavanshi’s study [44]. 

Further studies have shown that, in healthy controls, *Oxalobacter* presence is associated with a complex network of bacteria, that may exhibit oxalate-degrading capacity themselves or exert a permissive role on the metabolic activity of *Oxalobacter* [48,49]. These networks are someway less represented in calcium stone formers [47,49] and, most of all, in stone formers not harboring *Oxalobacter* in their fecal microbiota [48]. Therefore, the oxalate degrading capacity of the intestinal microbiota relies on a complex ecosystem and not solely on *Oxalobacter*, as believed before the emergence of high throughput sequencing techniques of the microbiota [7]. This concept has been confirmed in mice transplanted with human feces colonized by *Oxalobacter*, where the transplantation procedure resulted in selective expansion of the network of bacteria related to *Oxalobacter* [52]. 

Recently, Zampini et al. found that urine samples of stone formers exhibit a local microbiota, which is only minimally related to the intestinal microbiota, unrelated to the presence of urinary tract infection and composed of taxa with low pathogenic potential [50]. Compared with controls, stone formers have a different composition of urinary microbiota, and particularly exhibit depletion of *Lactobacillus* [50]. In around 20% of stone formers, next-generation sequencing techniques also allowed to clearly identify a stone microbiota, mainly composed of members of the genera *Staphylococcus, Enterobacter, Escherichia,* and *Lactobacillus* and with a different composition than the urinary microbiota [51]. Interestingly, the presence of these potentially pathogenic taxa was not associated with clinically evident infections [51]. 

## 3. The Role of Diet in the Gut-Kidney Axis 

### 3.1. The Determinants of Nephrolithiasis-Associated Gut Microbiota Dysbiosis

It has been postulated that the nephrolithiasis-associated gut and urinary microbiota dysbiosis could depend on increased exposure to antibiotic therapies [53]. This hypothesis is supported by two large population-based epidemiologic studies, showing that lifetime exposure to antibiotics, and particularly long-course treatments occurring during the younger age, are associated with increased risk of developing kidney stone disease [54,55]. In 25,981 patients with nephrolithiasis and 259,797 controls, the adjusted odds ratios for kidney stone disease ranged from 1.27 (95% CI 1.18–1.36) to 2.33 (95% CI 2.19–2.48) for prescription in the 12 months before assessment of broad-spectrum penicillins and sulfonamides, respectively [54]. Intermediate odds ratio values were found for cephalosporins, fluoroquinolones, and nitrofurantoin, irrespective of the reason for prescription [54]. In 5010 females participating to the Nurses’ Health Study I and II, cumulative use of antibiotics for 2 or more months in the age ranges of 40 to 49 and 40 to 59 were significantly associated with a higher risk of developing incident kidney stones (pooled hazard ratios 1.48, 95% CI 1.12–1.96, and 1.36, 95% CI 1.00–1.84, respectively) [55]. 

Although prolonged antibiotic exposure is able to disrupt intestinal microbiota composition inducing long-lasting alterations, it represents just one among a plethora of environmental factors associated with gut microbiota composition [56]. The metagenome-wide association study conducted in a Dutch population of 1135 subjects by Zhernakova and colleagues has shown that several factors related to lifestyle, diet, diseases, and drugs are associated with inter-individual differences of gut microbiota composition [57]. Among these factors, dietary factors represent the longest and most complex list [57]. 

In spite of this, a nutritional investigation has been comprehensively performed in only two studies comparing the fecal microbiota composition between stone formers and controls [47,49]. Stone formers generally have higher salt and animal protein intake, and lower calcium and FAV intake than stone-free controls [58,59,60]. The differences in gut microbiota composition between stone formers and controls may thus, at least partly, depend on different dietary habits, and nutrition could represent one of the main forces driving the so-called “gut-kidney axis” in kidney stone disease. 

Although no studies have specifically focused on this topic to date, there is much evidence showing that the dietary alterations associated with nephrolithiasis have the potential of influencing the microbiota composition. 

### 3.2. Salt and Microbiota 

High salt intake has been considered one of the main nutritional imbalances favoring calcium stone formation, especially through an increase in urinary calcium excretion and a decrease of urinary excretion of lithogenesis inhibitors, such as citrate [61]. Dietary salt restriction is significantly associated with a reduction of urinary calcium excretion and the prevention of recurrences in idiopathic calcium stone formers [62,63]. 

Salt has been used as a popular cure for centuries due to its antimicrobial properties [64]. However, the effects of salt intake on gut microbiota have been investigated only in very recent times. In the Dietary Approaches to Stop Hypertension (DASH)-Sodium Feeding Study, Derkach and colleagues showed that in 119 patients at high risk for hypertension, different levels of salt intake were associated with different urinary levels of several metabolites, including some of gut microbial origin [65]. Namely, high-salt intake was associated with decreased urinary levels of compounds related to fatty acid, benzoate, indole, isovalerate, methionine, and tryptophan metabolism and of the microbial metabolites 4-ethylphenylsulfate and 4-hydroxyphenylpiruvate [65]. 

The hypothesis that salt intake can modulate gut microbiota composition has been later confirmed in animal studies [66,67,68,69]. The administration of 2% NaCl in drinking water to mice resulted in the induction of gut microbiota dysbiosis, the elevation of gut mucosa permeability, and translocation of gut bacteria into the kidney, with the induction of hypertension and renal injury [66]. The strongest effect of a high-salt diet on mouse microbiota was the depletion of *Lactobacillus.* This alteration was also associated with the induction of T helper 17 cells, potentially contributing to hypertension by sustaining autoimmunity [67]. The high-salt diet was also associated with increased murine plasma concentrations of trimethylamine N-oxide (TMAO), an emerging marker of cardiovascular disease produced by the gut microbiota [68], and altered morphology of murine intestinal villi and crypts [69]. Interestingly, the administration of a probiotic blend containing *Lactobacillus* or a betaine-based prebiotic supplement was able to almost completely counteract the detrimental consequences of a high-salt diet for murine microbiota diversity and blood pressure [67,69]. 

To date, the effects of a high-salt diet on the human microbiota composition and functionality have been investigated in only two studies. A moderate 14-day salt challenge caused a significant reduction of the representation of *Lactobacillus* in a small group of volunteers [67]. In a large group of subjects of multi-ethnic origin and different geographical provenience, the fecal salinity was significantly associated with decreased microbiota diversity, depletion of bacteria with purported health-promoting activity, such as *Bifidobacterium* and *Akkermansia muciniphila*, and increased representation of halophilic bacterial and archeal species [70]. These results support the hypothesis that the gut microbiota is actively involved in the pathophysiology of salt-sensitive hypertension and modulates cardiovascular risk [71]. The relationship between salt and gut microbiota has not been investigated in kidney stone formers yet. However, the mentioned studies support the hypothesis that nephrolithiasis-associated gut microbiota dysbiosis is at least partly dependent on dietary salt intake. 

### 3.3. Animal Proteins and Microbiota

High-protein intake, particularly of animal origin, is considered an important risk factor for calcium nephrolithiasis. Animal proteins raise the renal acid load, which is associated with reduced excretion of lithogenesis inhibitors such as citrate [72,73] and incident kidney stones in population-based studies [74]. Amino acid and protein supplementation are also associated with increased urinary calcium excretion [75,76], while mild protein intake restriction reduces calcium excretion [77]. Thus, limiting animal protein intake is one of the cornerstone measures for preventing calcium lithiasis recurrence [16,17,62]. In the only rigorous randomized controlled trial on dietary prevention of calcium nephrolithiasis published to date, balanced animal protein intake was part of the dietary strategy that proved more effective in reducing recurrence of renal colic after a 5-year follow-up, compared with the control low-calcium diet [62]. 

The relationship between protein intake and gut microbiota composition has been investigated in several animal studies, giving conflicting results [78,79,80,81,82,83,84,85]. Rats fed a high-protein diet exhibited pro-inflammatory changes in gut microbiota composition, with an overrepresentation of pathobionts, such as *Escherichia*/*Shigella* and *Enterococcus*, depletion of species associated with the synthesis of short-chain fatty acids (SCFAs), such as *Faecalibacterium*, and protection of mucosa, such as *Akkermansia* [78,79]. These alterations of gut microbiota composition were emphasized when proteins were of animal origin [80]. However, other studies have shown that the intake of protein, especially of chicken origin, may also be associated with positive changes in gut microbiota composition of rats, including overgrowth of *Akkermansia* [81], *Lactobacillus* [82], and SCFA-producing taxa [83]. Red meat intake was also associated with increased representation of *Lactobacillus* and increased biodiversity in two studies [84,85], but in one of them, these changes were also accompanied by depletion of *Prevotella* and SCFA producers [85]. 

A recent experimental study has demonstrated that the impact of protein intake on gut microbiota composition of mice may depend on the absolute amount of proteins, with the highest representation of beneficial taxa, such as SCFA-producing genera, for moderate intake, and lowest representation for very high or very low intakes [86]. Therefore, the relationship between protein intake and microbiota composition may be U-shaped. However, the influence of nutritional intakes of other macronutrients, and particularly lipids, may be stronger than that of proteins for shaping the mouse microbiota composition [87]. 

Human studies in this field have been recently reviewed in a position paper on high-protein diets by the My New Gut Study Group [88]. In summary, these studies highlight that high-protein diets with unrestricted calories and fibers are associated with increased representation of bile-tolerant bacteria (*Alistipes*, *Bacteroides*, *Bilophila*) and decreased representation of Firmicutes, *Bifidobacterium* and *Roseburia* [89]. However, high-protein diets may also increase the microbiota abundance of *Faecalibacterium prausnitizii*, which is generally considered a health-promoting species due to its capacity to produce SCFA [90]. When diets include fixed amounts of calories and fibers, quantitative and qualitative variations of protein intake were not able to significantly modify gut microbiota composition, but modulated bacterial metabolism towards different metabotypes [91,92]. 

In this perspective, the microbiota composition and metabolic function are much more dependent on the overall nutritional pattern than on the intake of a single nutrient. Adherence to a Mediterranean-style diet was associated with beneficial effects in gut microbiota composition in two distinct studies [93,94]. In both studies, high animal protein intake was instead associated with the reduced representation of *Prevotella* and SCFA producers and increased representation of *Ruminococcus* [93,94]. Interestingly, reduced adherence to a Mediterranean-style diet is associated with an increased risk of incident nephrolithiasis in a large population-based study conducted in Spain [95]. Moreover, reduced adherence to the DASH-style diet (i.e., high salt and high animal protein intake) was associated with increased risk of kidney stones due to pro-lithogenic urine chemistry in three US cohorts [96,97]. 

Therefore, protein intake could represent, together with the overall dietary pattern, one of the main elements driving the alterations of gut microbiota composition detected in calcium stone formers. 

### 3.4. Oxalate Intake and Microbiota

Human oxalate metabolism is rather complex, and only marginally depends on dietary oxalate intake [5,98]. Intestinal oxalate absorption is, in fact, much more influenced by the calcium/oxalate ratio in the diet than by absolute oxalate intake [98,99]. Moreover, a consistent fraction of urinary oxalate is of endogenous origin as a product of hydroxyproline and ascorbic acid catabolism [98,100]. Dietary intake of these substances also has some part in determining urinary oxalate excretion [5,98,100]. Finally, hyperoxaluria, a urinary metabolic abnormality frequently found in calcium oxalate stone formers, is often caused by active oxalate secretion in kidney tubules [101]. 

In this scenario, a high dietary oxalate intake is associated with only a mild increase in the risk of kidney stones, although in some cases, this risk may retain great clinical relevance [102]. Reduction of oxalate intake can paradoxically increase urinary oxalate excretion and stone risk if it is not associated with other dietary measures [103,104]. Reducing oxalate intake is, therefore, indicated for preventing recurrences only in the case of mild hyperoxaluria [16,17]. Moreover, the reduction of indirect dietary sources of oxalate, such as animal proteins or ascorbic acid, is generally more effective than a low-oxalate diet for reducing oxaluria and preventing calcium oxalate stones [105,106]. 

In spite of these clinical concepts, dietary oxalate intake seems to be a powerful modulator of gut microbiota composition. In the last 5 years, Miller and colleagues have shown that the intestinal microbiota of the mammalian herbivore *Neotoma albigula* has an extremely high capacity of oxalate degradation regardless of oxalate intake levels [107,108,109]. In fact, the microbial ecosystem harbored in the gastrointestinal system of this rat shows several adaptive changes to increasing levels of oxalate intake [108]. In this ecosystem, oxalate-degrading capacities rely on several species, including *Lactobacillus, Enterococcus*, and *Clostridium*, either with a direct or a permissive role on oxalate degradation [107]. Dietary oxalate challenge resulted in an increased representation of 117 taxa within the microbial ecosystem, including the well-known *Oxalobacter* [109]. Interestingly, many of these taxa were found to be significantly depleted, compared with controls, in a group of human stone formers (Table 3) [49]. 

Adaptive changes of gut microbiota composition in stone formers following a high-oxalate diet could also help to explain why, in the Suryavanshi study mentioned above [44], stone formers had a high representation of oxalate-degrading species, a finding that is not coherent with the other studies listed in Table 3. A high intake of foods with elevated oxalate content, such as almonds, hazelnuts, walnuts, and pistachios, has been associated with specific changes of gut microbiota composition [110,111,112,113,114,115]. These changes are generally considered beneficial for human health and include an increased relative abundance of *Lachnospira, Roseburia, Dialister, Faecalibacterium,* and *Lactobacillus* with increased production of SCFA [110,111,112,113,114,115]. The effects on Bifidobacteria and non-pathogenic Clostridia were uncertain, with some studies reporting increased representation and others reporting depletion [111,113]. 

The role of oxalate in inducing these microbiota modifications is, however, uncertain. Nuts, and particularly the hazelnut skin, contain a high amount of polyphenol compounds that interact with the microbiota, are metabolized at this level and can shape the composition of microbial communities [116]. The effect of the intake of foods with high oxalate content on the gut microbiota may also depend on the pre-existing microbiota metabotype. Haaskjold and colleagues recently reported the absence of oxalate-degrading capacity in the gut microbiota as the main cause of renal failure in a patient who ate extremely high amounts of almonds, corresponding to a dietary oxalate challenge [117]. Therefore, the interaction between dietary oxalate, gut microbiota, and lithogenic risk may be extremely complex and needs further investigation in the future. 

### 3.5. Calcium Intake and Microbiota

Regular consumption of foods with high calcium content, either from dairy or non-dairy sources, is known to be protective against the formation of calcium kidney stones, while consumption of calcium supplements should be discouraged in stone formers [14,15,118]. Restriction of dietary calcium intake proved less effective than salt and protein restriction in preventing kidney stone recurrence [62]. However, the effect of dietary calcium on stone-forming propensity also depends on the oxalate content of the diet (i.e., the calcium/oxalate balance) and on the timing of calcium consumption [99,119,120,121]. In fact, stone formers have a higher fractional intestinal calcium absorption than subjects not suffering from nephrolithiasis, and foods with high calcium content should be consumed during balanced meals to protect against the risk of hypercalciuria [122,123,124]. 

To date, little is known about the relationship between dietary calcium intake and gut microbiota. However, one study conducted in mouse models of obesity has highlighted that the introduction of calcium supplementation could be associated with beneficial effects on the gut microbiota [125]. Reduced or excessive calcium intake could also influence the development of obesity through modulation of microbiota in weaning mouse pups [126]. Moreover, modulation of microbiota through prebiotic supplements can positively modulate dietary calcium absorption in mice, which represents a promising strategy for reducing the burden of hypercalciuria in kidney stone formers [127]. 

### 3.6. FAV Intake, legume Intake, and Microbiota

A high FAV intake is associated with reduced risk of incident kidney stones [14,15,128] and with a reduction of lithogenic potential in urine chemistry [129]. Thus, increasing FAV intake is regarded as one of the main non-pharmacologic prescriptions for reducing the risk of kidney stone recurrence [16,17]. A good FAV intake is in fact able to raise the urinary volume, excretion of inhibitors of lithogenesis, such as citrate, potassium, and magnesium, and reduce the renal acid load [129]. 

A high intake of legumes is also generally regarded as protective against lithogenesis, due to the inhibitory effect of inositol hexaphosphate on urinary crystallization phenomena [130,131]. However, some legumes also have a moderate-to-high content in oxalate [132], which may be responsible for increased risk of lithogenesis observed with high legume intake in some reports [133]. 

Fibers, that is, non-digestible carbohydrates found exclusively in plants, are the main components of FAV interacting with the microbiota. These compounds can in fact be metabolized by several microbial species and represent the main substrate for the synthesis of SCFA (acetate, butyrate, propionate) by the microbiota. Several studies indicate that high fiber intake is also able to modulate the gut microbiota composition towards an increase of representation of SCFA-producing species, lactic acid bacteria, and species with purported health-promoting actions, including *Faecalibacterium, Bifidobacterium*, and *Lactobacillus*, at the expense of a reduction of pathobionts [134,135,136]. These differences also emerged when comparing subjects who follow a Mediterranean-style diet with subjects who follow a Western-style diet [93,94]. 

Soluble fiber supplementation resulted in increased biodiversity of gut microbiota composition, increased stability over time, and increased representation of Bifidobacteria [137,138]. Interestingly, an increase in fiber intake was also associated with decreased levels of *Oxalobacteraceae*, probably as a consequence of the reduced oxalate content of ingested foods [138]. 

The effects of insoluble fiber intake on gut microbiota composition are less known and generally considered negligible in comparison with that of soluble fibers such as inulin [139]. However, one study showed that the supply of different types of fibers, such as insoluble pectin vs. soluble inulin, resulted in the selective promotion of growth of different microbial communities in pH-controlled continuous-flow fermentors containing microbiota of human gut origin [134]. Moreover, a comparison of dietary habits and microbiota composition between children from Europe and rural areas of Burkina Faso showed that the high insoluble fiber intake of Burkinabe participants was associated with selective expansion of taxa, including *Xylanibacter* and *Prevotella*, harboring genes for cellulose and xylan hydrolysis that were completely absent in European counterparts [140]. Thus, the effects of FAV intake on the microbiota could also depend on the ratio between insoluble and soluble fibers of ingested foods. 

In obese subjects, increasing FAV intake is associated with reduced weight gain, more favorable body composition, and different urinary metabolic profile compared with obese subjects who follow their usual diet [141,142,143]. All these physiological modifications are mediated by the gut microbiota [141,142,143]. High FAV intake is in fact, associated with microbial metabolism of plant flavonoids, resulting in the systemic absorption of several compounds acting as metabolic modulators [142,144]. One of these compounds is hippuric acid, whose urinary excretion is considered a marker of FAV intake of potential utility in kidney stone formers for monitoring adherence to dietary recommendations [145]. 

Dietary supplementation with fruit juices that may have positive effects in modulating lithogenesis of kidney stone formers [146] is also associated with increased gut microbiota biodiversity and representation of taxa with health-promoting activities, such as Bifidobacteria [147,148,149,150]. Consumption of fruit juices is associated with increased fecal and urinary levels of metabolites of microbial origin exhibiting favorable metabolic activities [150,151]. 

Legume intake can favorably affect the microbiota composition in terms of increased representation of Bifidobacteria and Lactobacilli and reduced Firmicutes/Bacteroidetes ratio [152]. This assumption is supported by human studies where soy or derivatives were administered to small samples of healthy subjects [152] and also by animal studies with dietary supplementation of soy or lentils [153,154]. However, these effects are probably not mediated by fibers, and depend on isoflavone-derived compounds that can be found in high amounts in legumes, and particularly in soy [155]. 

Although not specifically focused on kidney stones, the current literature supports the hypothesis that the beneficial effects of increasing FAV or juice intake on the risk of kidney stone disease may be at least in part mediated by changes in gut microbiota composition and metabolic function. 

### 3.7. Water Intake and Gut Microbiota

Poor hydration is a fundamental risk factor for kidney stone disease, and daily water intake >2 L represents a cornerstone measure for preventing recurrences [146]. The mechanisms that link water intake with lithogenesis are well-known and largely dependent on physicochemical factors and renal physiology [146]. However, recent studies suggest that hydration, and the type of drunk water, may also influence the gut microbiota. Therefore, water intake could also influence lithogenesis through the gut-kidney axis. 

Water pH is able to influence the microbiota composition of mice, probably because different pH allows the growth of different microbial populations in drinking water [156]. The supply of acidified drinking water to mice was able to induce deep changes of gut microbiota composition, including the overrepresentation of several taxa that are notable components of microbiota in humans, such as *Bacteroides*, *Alistipes*, *Barnesiella*, and *Lactobacillus* [157]. For this reason, some authors recently proposed that drinking water pH should be considered as a covariate in microbiome studies conducted in animal models [158]. 

These surprising findings are also supported by two human studies [159,160]. Murakami et al. found that regular consumption of alkaline water is associated with significant changes in gut microbiota composition, namely an increase of representation of Christensenellaceae, Bifidobacteriaceae, and Oxalobacteraceae [159]. Hansen et al. reported no effect of drinking water pH on microbiota biodiversity assessed by the Shannon index but showed significant changes in the abundance of some taxa, including *Ruminococcaceae* and *Prevotella copri* after the ingestion of water with a neutral pH [160]. 

However, these animal and human studies do not fully consider the mineral composition of drinking water and the effect of diet on the microbiota. Thus, their conclusions should be interpreted with much caution, and the relationship between the composition of drinking water and gut microbiota composition needs further investigation in the future. 

## 4. Conclusions and Perspectives

**Figure 1:**
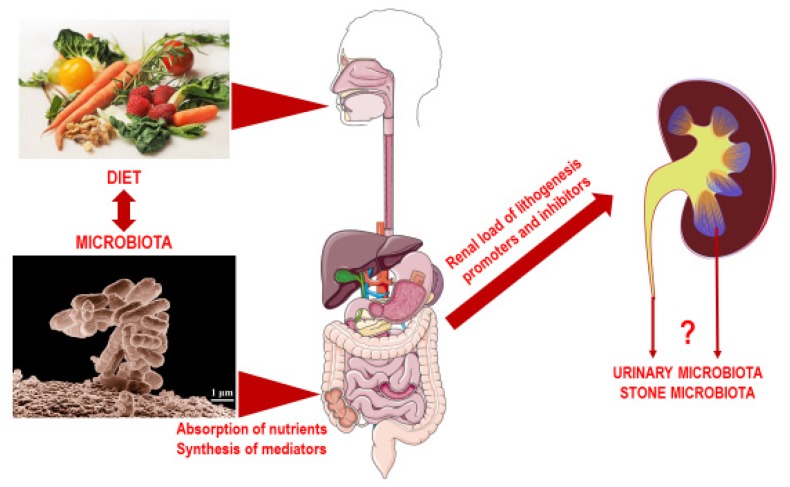
Representation of the possible role of nutrition in the gut-kidney axis in nephrolithiasis.

Several studies support the hypothesis that the intestinal microbiota composition is able to influence lithogenesis beyond the simple presence or absence of *Oxalobacter formigenes*. At the same time, all the main nutritional imbalances associated with increased risk of calcium nephrolithiasis are associated with specific alterations of gut microbiota composition (Table 4). Although the relevance of these changes for kidney stone pathophysiology is still unclear, since no study has comprehensively evaluated the gut-kidney axis from a nutritional perspective, they allow the hypothesis that the microbiota acts as a metabolic modulator at the cross-road between nutrition and kidney function, influencing the lithogenic risk (Figure 1). 

Future studies on the gut-kidney axis in nephrolithiasis should not be limited to the description of fecal microbiota composition and comparison with healthy controls anymore, but should also embed thorough nutritional investigation and functional aspects of the interaction between nutrients and the microbiota. The nutritional intervention targeted at manipulating the microbiota composition and function is a promising field for modulating lithogenic risk and identifying novel strategies for the prevention of nephrolithiasis recurrences. Moreover, the role of urinary and stone microbiota (Figure 1) is another controversial point that should be investigated in the future to identify whether it is actively involved in kidney stone formation and represents another possible target for novel therapeutic strategies. 

## Figures and Tables

**Table 1:** Overview of human observational studies investigating the association between nephrolithiasis and prevalence of *Oxalobacter formigenes* in feces.

AUTHOR, YEAR [REF]	METHODS OF MICROBIOTA ANALYSIS	PARTICIPANTS	MAIN RESULTS	NOTES
Sidhu H et al., 1998 [26]	Culture + PCR if cultures negative	43 children with cystic fibrosis, 21 healthy children	Prevalence of *Oxalobacter* 16% in patients and 71% in controls; patients without *Oxalobacter* had hyperoxaluria and high stone risk	None of the participants had kidney stones.
Sidhu H et al. 1999 [27]	Culture + PCR	51 adult idiopathic calcium oxalate SFs, 44 healthy volunteers	Prevalence of *Oxalobacter*: - 75% in controls - 80% in first time stone formers - 38% in recurrent stone formers - 13% in highly recurrent stone formers	Cases and controls inhomogeneous for age and geographical location.
Kumar R, et al. 2004 [28]	PCR	37 ulcerative colitis, 11 Crohn’s disease, 87 calcium SFs, 48 healthy controls	Prevalence of *Oxalobacter*: 10% in IBD, 29% in stone formers, 56% in controls. Oxalate excretion significantly higher in those without *Oxalobacter* colonization	The study focused on IBD-associated forms of calcium stones.
Kaufman DW, et al. 2008 [29]	Culture	247 calcium SFs, 259 age-, sex- and location-matched controls	Prevalence of *Oxalobacter*: 17% in stone formers, 38% in controls. No association between *Oxalobacter* and oxalate excretion.	Absence of genomic methods of *Oxalobacter* detection
Siener R, et al. 2013 [32]	Culture + PCR	37 calcium SFs	Prevalence of *Oxalobacter*: 30%. In colonized subjects, oxalate excretion is lower only under controlled dietary oxalate intake.	Study focused on oxalate metabolism; no controls enrolled.
Tavasoli S, et al. 2020 [30]	PCR	29 SFs with hyperoxaluria, 29 SFs without hyperoxaluria, 29 controls	*Oxalobacter* more prevalent and abundant in controls and inversely related to oxaluria	Investigated also *Oxalobacter* abundance in feces

PCR = Polymerase Chain Reaction; IBD = Inflammatory Bowel Disease; SFs = Stone Formers.

**Table 2:** Overview of human intervention studies investigating the effects of the administration of oxalate-degrading bacteria on lithogenic risk.

AUTHOR, YEAR [REF]	PROBIOTIC	DESIGN, PARTICIPANTS AND FOLLOW-UP DURATION	KEY FINDINGS
Campieri C, et al. 2001 [36]	Lactobacilli (*L. acidophilus*, *L. plantarum*, *L. brevis*) + *Bifidobacterium infantis*	Prospective single-arm intervention, 6 calcium stone formers, 4-week of follow-up	All participants experienced redution of urinary oxalate excretion (average 40%)
Duncan SH, et al. 2002 [37]	*Oxalobacter formigenes* strain HC1 isolated from human feces	Prospective single-arm intervention, 2 healthy volunteers, 6-h follow-up	Decrease of urinary oxalate excretion after a dietary oxalate load following the probiotic administration.
Lieske JC, et al. 2005 [38]	Oxadrop® (*L. acidophilus*, *L. brevis*, *S. thermophilus*, *B. infantis*)	Prospective single-arm intervention, 10 stone formers with intestinal malabsorption, 1-month follow-up	Decrease of urinary oxalate excretion shown in 7 participants over 10 (average effect size: −19%).
Goldfarb DS, et al. 2007 [39]	Oxadrop® (*L. acidophilus*, *L. brevis*, *S. thermophilus*, *B. infantis*)	RCT, 20 calcium oxalate stone formers with hyperoxaluria, 4-week follow-up	No significant variation of urinary oxalate excretion after treatment in both intervention and control arm.
Okombo J, et al. 2010 [40]	VSL#3 (*L. acidophilus*, *L. gasseri, B. lactis*)	Prospective single-arm intervention, 11 stone-free volunteers, 4-week follow-up	Reduction of fractional oxalate absorption after a dietary oxalate load (from 31% to 12%).
Hoppe B, et al. 2011 [41]	Oxabact® (*Oxalobacter formigenes*)	RCT, 42 adolescents with primary hyperoxaluria, 24-week follow-up	Reduction of urinary oxalate excretion in both intervention and control arm (average effect size 20% in both groups)
Al-Wahsh I, et al. 2012 [42]	VSL#3 (*L. acidophilus*, L. *gasseri*, *B. lactis*)	Prospective single-arm intervention, 11 healthy stone-free volunteers, 24-h follow-up	Reduction of urinary oxalate excretion after a standardized dietary oxalate load
Siener R, et al. 2013 [43]	Oxadrop® (*L. acidophilus*, *L. brevis*, *S. thermophilus*, *B. infantis*)	Randomized cross-over trial, 20 stone-free healthy volunteers under high-oxalate diet, 5 week follow-up	No significant variation of oxaluria detected.

RCT = Randomized Controlled Trial.

**Table 3:** Overview of human studies investigating the fecal or urinary microbiota composition in kidney stone formers by using next-generation sequencing techniques.

AUTHOR, YEAR [REF]	PARTICIPANTS	STONE TYPES	COUNTRY	SAMPLES	MAIN FINDINGS IN STONE FORMERS	TAXA DEPLETED IN STONE FORMERS
Suryavanshi et al., 2016 [44]	24 recurrent KSF 15 controls	Calcium oxalate	India	Feces	Gut microbiota dysbiosis with different clusterization of composition and functionality. Urinary oxalate excretion correlated with the abundance of 12 taxa. Several taxa harboring oxalate-degrading functionalities identified in both KSF and controls.	Several species, including *Faecalibacterium prausnitzii*
Stern et al., 2016 [45]	23 KSF 6 controls	Calcium Uric acid	United States	Feces	Different microbiome composition with the prevalence of *Bacteroides* over *Prevotella.*	*Prevotella*
Tang et al., 2018 [46]	13 multiple KSF 13 controls	Radio-opaque	China	Feces	Trend towards reduced biodiversity. Different microbiome composition clusters between KSF and controls.	*Eubacterium, Dorea, Ruminiclostridium, Anaerostipes, Fusicatenibacter, Subdoligranulum, Holdemania, Dialister, Ruminococcus, Parasutterella, Bilophila*
Ticinesi et al., 2018 [47]	52 recurrent KSF 48 controls	Calcium	Italy	Feces	Reduced fecal microbiota biodiversity. Separate clusterization of KSF and controls. Urinary oxalate excretion correlated with the abundance of 5 taxa. Reduced representation of bacterial genes involved in oxalate degradation. Oxalate-degrading functionalities harbored in several species.	*Dorea, Enterobacter, Faecalibacterium prausnitzii*
Suryavanshi et al., 2018 [48]	24 recurrent KSF 48 controls	Calcium oxalate	India	Feces	Dysbiosis not limited to eubacteria and also involving archaea and eukaryotes. Species able to metabolize oxalate and produce butyrate were depleted in KSF not colonized with *Oxalobacter*.	Several species with oxalate-metabolizing properties and butyrate producers, including *Prevotella* and *Ruminococcus*
Miller et al., 2019 [49]	17 KSF 17 controls	Calcium oxalate Uric acid Struvite Cystine	Canada	Feces	KSF has reduced representation of a network of bacteria directly involved in oxalate degradation or co-occurring with *Oxalobacter* in network analysis. These taxa include those stimulated by oxalate intake in rodent models.	103 bacterial taxa, including *Ruminococcus* and *Oscillospira*
Zampini et al., 2019 [50]	24 KSF43 controls	Calcium Uric acid Mixed calcium+ uric acid	United States	Feces Urine Stones	Fecal microbiota similar in KSF and controls. Urinary microbiota had composition independent of stool microbiota. KSF had urinary microbiota dysbiosis correlated with antibiotic treatments, sex and family history. Stones harbor an independent microbiota population.	*Lactobacillus* underrepresented in urinary samples
Dornbier et al., 2019 [51]	52 KSF	Any composition	United States	Urine Stones	In 20% of KSF, stone samples exhibit microbial communities with a composition independent of urine. Main components of these communities: *Staphylococcus, Veillonella, Streptococcus, Enterobacter, Escherichia.*	No comparison with controls provided in the study

KSF = Kidney Stone Formers.

**Table 4:** Overview of the main nutritional imbalances associated with high lithogenic risk and their effects on gut microbiota composition.

NUTRITIONAL IMBALANCE	EFFECT ON URINE CHEMISTRY AND LITHOGENIC RISK	EFFECT ON GUT MICROBIOTA COMPOSITION
High salt intake	Increase in urine calcium Decrease in urine citrate Increased risk of CaOx lithiasis	Depletion of *Lactobacillus, Akkermansia, Bifidobacterium*
High animal protein intake	Increase in urine calcium Increase in urine uric acid Decrease in urine pH Increase of renal acid load Increased risk of CaOx and AcUr lithiasis	Depletion of Firmicutes, *Bifidobacterium, Roseburia, Prevotella* Increased representation of bile-tolerant bacteria Increased representation of *Faecalibacterium*
High oxalate intake	Increase in urine oxalate Mild increased risk of CaOx lithiasis	Expansion of oxalate-degrading species Increased representation of *Lachnospira, Roseburia, Dialister, Faecalibacterium* and *Lactobacillus* (probably due to other nutrients contained in oxalate-rich foods)
Low calcium intake	Increase in urine oxalate Increase in urine calcium from bones Increased risk of CaOx lithiasis	Reduced biodiversity with depletion of species producing SCFAs (in mice)
Low FAV intake	Decrease in urinary inhibitors of lithogenesis Decrease in urinary volume	Depletion of lactic acid bacteria Depletion of species producing SCFAs Depletion of *Bifidobacterium, Faecalibacterium, Lactobacillus*
Poor hydration	Decrease in urinary volume	Alterations of representation of some selected taxa, including *Ruminococcaceae, Prevotella*, Bifidobacteriaceae and Oxalobacteriaceae depending on the pH of ingested fluids

CaOx = Calcium Oxalate; AcUr = Uric Acid; SCFAs = Short-Chain Fatty Acids; FAV = Fruit and Vegetables.
